# A Fast Linearly Wavelength Step-Swept Light Source Based on Recirculating Frequency Shifter and Its Application to FBG Sensor Interrogation

**DOI:** 10.3390/s19030593

**Published:** 2019-01-30

**Authors:** Quan Yuan, Zhaoying Wang, Lipei Song, Zhaoyu Lu, Diannan Hu, Jiaqi Qin, Tianxin Yang

**Affiliations:** 1 Key Laboratory of the Ministry of Education on Optoelectronic Information Technology, School of Precision Instrument and Optoelectronics Engineering, Tianjin University, Tianjin 300072, China; ewanyuan@tju.edu.cn (Q.Y.); luzhaoyu@tju.edu.cn (Z.L.); diannanhu@tju.edu.cn (D.H.); jiaqi_qin@tju.edu.cn (J.Q.); tyang@tju.edu.cn (T.Y.); 2 Institute of Modern Optics, Nankai University, Tianjin 300350, China; gm_imo@nankai.edu.cn

**Keywords:** wavelength step-swept source, wavelength-to-time mapping, fiber Bragg grating, sensor interrogation

## Abstract

A wavelength step-swept light source (WSSL) using a recirculating frequency shifter loop (RFSL) based on a single-side-band (SSB) modulator is proposed, in order to achieve a linear and fast wavelength-sweeping. The swept step can be tuned from 1.2 pm to 128 pm by adjusting a precise and stable radio frequency (RF) signal that is applied to the SSB modulator. The swept rate can be tuned up to 99 kHz in a range of over 5.12 nm. Wavelength-to-time mapping is used to measure static strain-induced or temperature-induced shifting of the reflected central wavelength of a fiber Bragg grating (FBG). Because of the high linearity of the light source, the interrogation linearity of the strain and the temperature are as high as 0.99944 and 0.99946, respectively. When a dynamic periodic strain applied to FBG sensor, the dynamic performance of the FBG sensor is successfully recorded in the time domain and its power spectral density of a fast Fourier transform (FFT) is calculated. The signal-to-noise ratio (SNR) of the power spectral density is over 40 dB for a 100 Hz dynamic strain and the calculated sensitivity is 0.048 με/Hz^1/2^. A sharp change in the strain frequency from 100 Hz to 500 Hz is captured in real time.

## 1. Introduction

Fiber Bragg grating (FBG) sensors are widely used in sensing fields, especially in strain and temperature measurements, because of their unique advantages, such as their small size, electromagnetic immunity, remote sensing capability, easy fabrication, etc. [1,2,3,4]. The fundamental basis for FBG sensors is the interrogation of the wavelength shift of the FBG, due to a change in the strain or temperature [5]. Several passive interrogation methods, such as optical interferometers [6,7], arrayed waveguide grating [8], passive optical filters [9,10], wavelength division coupler [11], have been reported to examine the change in Bragg wavelength using a broadband optical light source. However, these systems have the major limitations of low signal-to-noise ratio (SNR) and interrogation speed. Therefore, fast variations in strain cannot be detected in real-time with these systems.

A wavelength-swept laser (WSL) has been developed [12,13,14,15,16,17,18,19,20,21,22,23,24] as a promising optical source to overcome these problems in FBG interrogation because of its advantages in terms of speed, resolution, and SNR. In particular, a laser source with a high swept rate of over 10 kHz has a wide range of applications in real-time FBG sensor systems [19]. When a WSL is applied to interrogate a FBG sensor, the reflected sensing wavelengths exactly correspond to the detected pulse positions in time domain. It is necessary for the WSL to have a fast and linear sweep for improving the sensing modality. Most reported WSLs and commercial tunable lasers use a broadband gain medium and wavelength selected filters, such as a fiber Fabry–Perot tunable filter [12,13,14], tunable ratio optical coupler [15], polygon mirror-scanning filter [16], etc. To increase the repetition rate of a swept laser, the tunable filters need to be driven by sinusoidal signals rather than linear signals. The linearity between the wavelengths in wavelength domain and the pulse positions in time domain degrades significantly with the increase of the swept rate. Additional tools have to be used to eliminate the nonlinearity introduced by the mechanical swept mechanism. Some other WSL-based FBG interrogation systems which do not use a mechanical swept mechanism are demonstrated, including a Fourier domain mode-locked laser [17,18], a wavelength-swept laser based on dispersion tuning technique [19], and an active mode locking fiber laser [20]. However, due to the wavelength tuning inside the laser cavity, the linewidth of the output is on the nanometer scale and limits the resolution of the interrogation system. Several other internal modulated WSLs with a narrow linewidth output, such as a vertical cavity surface emitting laser [21] and a 1550-nm standard telecom distributed feedback (DFB) laser [22], have been proposed. The wavelength tuning is deduced from the dependence of the emission wavelength on the laser current. Thus, a characterization step of current calibration is required for a linear output, which makes the systems more complicated. In Reference [23], a linearized wavelength swept thermo-optic laser chip was proposed for an FBG interrogation system. Its linearity of wavelength-time fitting was 0.99994, which is the highest linearity currently reported to our knowledge. However, its wavelength sweeping rate was only 16 Hz in the swept range of 11.8 nm due to the slow thermo-optical effect. In Reference [24], a narrow line width tunable laser source was generated by driving an optical single-side-band (SSB) modulator using a linear chirp RF signal outside the cavity. However, the swept range was lower than 0.05 nm, which limited the dynamic range to 6 GHz. 

In this work, we demonstrate an FBG interrogation system using a wavelength step-swept light source (WSSL) based on a recirculating frequency shifter loop (RFSL) [25,26]. In our system, a linear mapping from wavelength to time is achieved, providing a simpler way to demodulate the FBG sensing signal in both static and dynamic FBG sensing interrogations. In the wavelength domain, the swept step of WSSL is precisely controlled by a radio frequency (RF) signal as a constant. In the time domain, the output pulse interval is determined by the length of RFSL, which is also a constant. Therefore, the wavelength-to-time mapping of the WSSL shows high linearity. In our experiments, the output of the WSSL showed a perfect linearity with an R-square value equal to 1. The swept step can be tuned from 1.2 pm to 128 pm by tuning the frequency of the RF signal (*f_RF_*). The swept rate can be up to 99 kHz with a swept range of over 5.12 nm. FBG interrogation of the static strain sensing and the temperature sensing based on the WSSL with different swept steps were achieved. The linearity of the strain sensing and the temperature sensing interrogation results were 0.99944 and 0.99946, respectively, with a swept step of 4.92 pm. With the swept rate of the WSSL at 40 kHz, dynamic strain sensing was interrogated. The calculated sensitivity was 0.048 με/Hz^1/2^ for a 100 Hz dynamic strain. The dynamic response of the FBG also successfully captured the sudden jump in the strain frequency from 100 Hz to 500 Hz.

## 2. Experimental Setup and the Performance of WSSL

**Figure 1:**
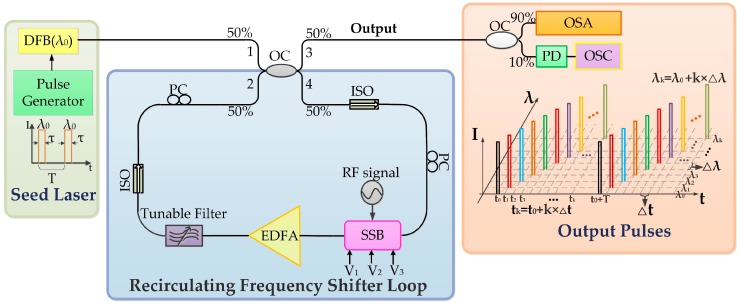
The principle setup of the wavelength step-swept laser source. DFB: Distributed feedback laser; PG: Pulse generator; OC: Optical coupler; ISO: Isolator; PC: Polarization controller; SSB: Single-side-band modulator; RF: Radio frequency; EDFA: Erbium-doped fiber amplifier; PD: Photodetector; OSA: Optical spectrum analyzer; OSC: Oscilloscope.

The setup of the WSSL is shown in Figure 1. The seed laser was generated by a pulse-modulated DFB laser with a central wavelength at 1547.52 nm (λ_0_) and the linewidth was less than 3 MHz. The temperature of the DFB laser was controlled at 25 °C ± 0.01 °C by a temperature control chip, which promised stability of the output wavelength of the seed DFB laser. The modulated pulses were square waveforms, of which pulse period (T) and pulse width (τ) were controlled by a digital pulse generator (Stanford DG535). In our experiments, τ was set to 100 ns. The seed laser beam was coupled into a recirculating frequency shifter loop. The loop included an optical coupler, two isolators (ISO) for ensuring unidirectional light propagation, a single-side-band (SSB) modulator (COVEGA Mach-40-086) driven by a RF signal for frequency shifting, an erbium-doped fiber amplifier (EDFA) for compensating the optical loss, a tunable filter for eliminating the amplified spontaneous emission (ASE) noise introduced by the EDFA and two polarization controllers (PC) for controlling the polarization state of the optical signal. The bandwidth of the tunable filter was set according to the swept steps and it determined the swept range of the WSSL. As the bandwidth of the tunable filter approached or exceeded the homogeneous broadening range (usually 6~7 nm) of erbium doped fiber (EDF), the ASE noise accumulated with circulation in the optical loop, and may form new frequency peaks offset from the original signal frequency [27]. Thus, the bandwidth of the optical filter should be set lower than the homogeneous broadening range of EDF. The output of the WSSL was sent to an optical spectrum analyzer (OSA, Anritsu MS9740A) and an oscilloscope (OSC, Tektronix 3052C) via a photodetector (PD).

The SSB modulator shifts the frequency of the seed signal by one swept step (*∆λ*) in each circulation [28]. The swept step (*∆λ*) is exactly determined by the frequency of the RF signal (*f_RF_*) which is expressed as:

(1) $$\Delta \lambda =f_{RF}\times \frac{\lambda^{2}}{c},$$

where *λ* is the central wavelength of the seed laser and *c* is the speed of light. Because *λ* changes in a range of several nanometers, *Δλ* changes approximately linearly to *f_RF_*. Driven signal *f_RF_* in our setup can be tuned from 150 MHz to 16 GHz and accordingly *∆λ* is tuned from 1.2 pm to 128 pm. As the phase noise of *f_RF_* (supplied by RF generator, Agilent E8257D) is lower than –134 dBc/Hz@20 kHz (at a 1-GHz carrier center frequency) in our experiments, the fluctuation of *∆λ* introduced by the phase noise can be neglected. For a specific value of *f_RF_*, the wavelength of the *k^th^* output pulse can be described as:

(2) $$\lambda_{k}=\lambda_{0}+k\times \Delta \lambda ,$$

The time-interval (*∆t*) of the output pulse is described as:

(3) $$\Delta t=\frac{n\times L}{\;c},$$

where *n* is the refractive index of the fiber and *L* is the length of the loop. In our system, *L* is about 50.4 m. Since the value of *L* is tens of meters and the swept range is several nanometers, the dispersion of the single mode fiber (typically 17 ps/(nm•km)) can be neglected in our system and *Δt* can be treated as a constant in the time domain. The output of the WSSL between the wavelength and the time domain is shown in Figure 1. 

The swept rate (*v*) of the laser source can be expressed as:

(4) $$v={\left(\frac{\Delta \Lambda }{\Delta \lambda }\times \Delta t\right)}^{-1}=\frac{\Delta \lambda }{\Delta \Lambda }\times \frac{c}{n}\times \frac{1}{L},$$

where *∆Λ* is the swept spectral range of the output pulses. It is clear that the swept rate can be increased proportionately by reducing the length of the recirculating frequency shifter loop (*L*).

**Figure 2:**
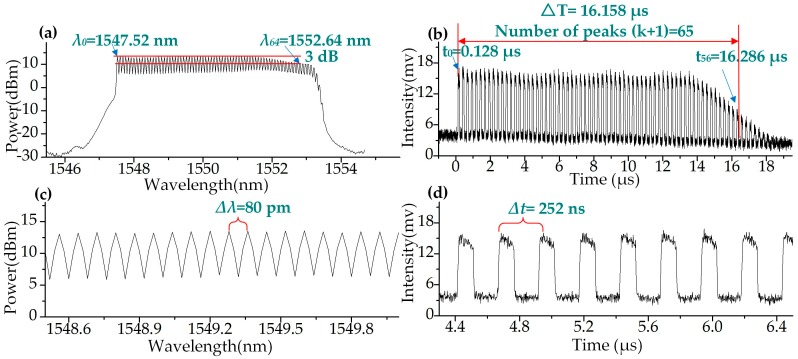
The spectrum and the waveform of the output of wavelength step-swept light source (*f_RF_* = 10 GHz). (**a**) Spectrum in wavelength domain. (**b**) The waveform in time domain. (**c**) The zoom-in figure of the spectrum. (**d**) The zoom-in figure of the waveform.

Different *f_RF_* were applied to the WSSL in the experiments. The spectrum and the waveform of the step-swept output pulses with *f_RF_* equal to 10 GHz (*∆λ* = 80 pm) are shown in Figure 2 as an example. The bandwidth of the filter was set to 5.8 nm. The 5.12 nm swept range from 1547.52 nm to 1552.64 nm was achieved with a 3 dB power flatness, as shown in Figure 2a. In total, 65 step-swept laser tones were detected. Figure 2b shows the step-swept pulses obtained in the time domain. The number of pulses was also 65, which agrees with that shown in Figure 2a. The gain spectrum of the EDFA we used was not flat enough, and the gain curve had a small dent at a wavelength of about 1552 nm, which caused the intensity and the SNR to decrease after a moment, at 14 μs (Figure 2b). The zoom-in figures of the spectrum and the waveform are shown in Figure 2c,d. The pulse interval was about 252 ns and the swept duration was 16.158 μs. The swept rate was 61.89 kHz. If the swept step is set at 128 pm, the swept rate can get to 99 kHz.

**Figure 3:**
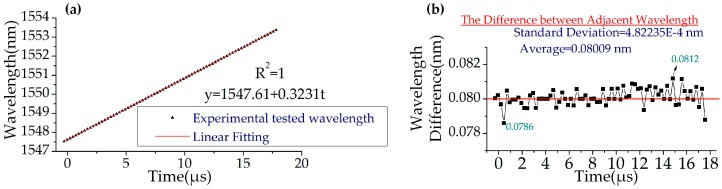
Further analysis of the linearity of the wavelength step-swept laser (*f_RF_* = 10 GHz). (**a**) The wavelength-time mapping of the wavelength step-swept source. (**b**) The wavelength differences between adjacent pulses.

Figure 3a shows the wavelength-to-time mapping of the results in Figure 2. A linear fit of the mapping gives an R-square value of 1, demonstrating the linearity of the WSSL. The wavelength differences between adjacent pulses are measured by the OSA and are shown in Figure 3b. The largest deviation is 0.0786 nm. The mean and the standard deviation are 0.08009 nm and 4.82235E-4 nm, respectively, which demonstrates the linearity of the WSSL.

As the wavelength sweeping is implemented outside of the seed DFB laser cavity, the linewidth of the output of the WSSL can remain at several MHz [29]. In addition, the single DFB laser can be replaced by a multi-wavelength seed source to achieve synchronous multi-wavelength sweeping, which can improve the swept range of the laser source without affecting the swept rate and linearity. 

## 3. Wavelength Interrogation of FBG Based on the WSSL

**Figure 4:**
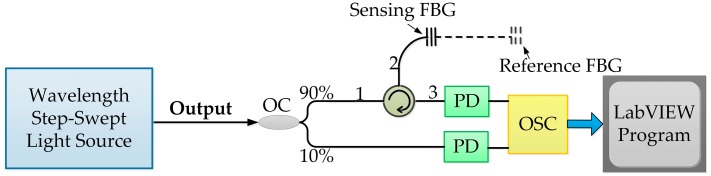
FBG interrogation system for the static strain sensing and the temperature sensing based on the WSSL. FBG: Fiber Bragg grating; PD: Photodetector; OSC: Oscilloscope.

In the experiments, we applied the wavelength step-swept laser source to a FBG sensing system. The experimental setup is shown in Figure 4. The output of the source was divided into two parts by a coupler: 10% of the output was sent into the OSC via a photodetector (PD); the other 90% output was fed into two FBG sensors (reference and sensing FBGs) by a circulator. The pulses reflected by the FBGs were sent into the OSC via a PD. The acquired data were processed using LabVIEW.

### 3.1. Wavelength Interrogation of Static Strain and Temperature Sensing

In the temperature sensing experiments, the sensing FBG was placed in a water bath. The central wavelength of the seed DFB laser was 1547.52 nm The Bragg wavelength of the sensing FBG was 1548.55 nm at 25 °C. The *f_RF_* applied to the SSB modulator were 2.5 GHz, 1.25 GHz, and 615 MHz respectively, so the swept steps were 20 pm, 10 pm, and 4.92 pm, correspondingly. The temperature of the water was changed from 21 °C to 81 °C in 3 °C increments.. The central wavelengths of FBG at different temperatures were calculated using LabVIEW, which adopts a noise filter algorithm to filter out low frequency noise, and a flatness error compensation algorithm to compensate for the unflatness of the power. 

**Figure 5:**
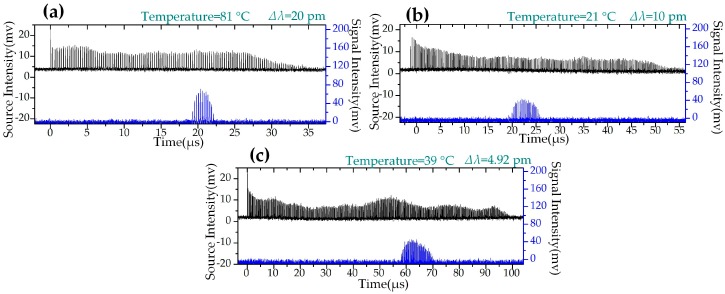
Waveforms of FBG temperature sensing based on WSSL with different sweep steps (∆λ). (**a**) ∆λ = 20 pm and the temperature is 81 °C. (**b**) ∆λ = 10 pm and the temperature is 21 °C. (**c**) ∆λ = 4.92 pm and the temperature is 39 °C.

**Figure 6:**
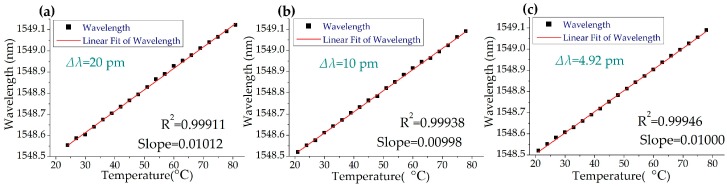
Interrogating results of FBG temperature sensing. (**a**) ∆λ = 20 pm. (**b**) ∆λ = 10 pm. (**c**) ∆λ = 4.92 pm.

Three experimental waveforms in the time domain are shown in Figure 5 as examples. In Figure 5, the black waveform is the original pulses and the blue waveform is the sensing pulses reflected by the sensing FBG. With decreasing swept steps, the resolution of interrogation theoretically increases. The interrogating results are shown in Figure 6; the central wavelength increases linearly with temperature. The slopes of the linear fitting are about 10.004 ± 0.1 pm/°C. The R-square values are above 0.9991. The slope and the R^2^ of the linear fitting at different swept steps are very close to each other, indicating that the laser source is able to provide good linearity between the wavelength and the temperature at different swept steps. In addition, the value of R^2^ increases slightly with the decrease in the swept step (∆λ) of the laser source, which indicates that smaller swept steps produce higher linearity.

In the static strain measurement experiments, the sensing FBG was placed at a uniform intensity cantilever, of which the deflection was proportional to the strain applied (66 μɛ/mm). In the experiments, the deflection changed linearly from 0 mm to 4.5 mm with an increment of 0.5 mm. The central wavelength of the seed DFB laser was 1547.52 nm. The Bragg wavelength of the sensing FBG was 1548.55 nm at 25 °C. Experiments with 20 pm, 10 pm and 4.92 pm swept steps were conducted respectively. To eliminate the interference of temperature fluctuation, the reference FBG (Bragg wavelength is 1549.02 nm at 25 °C) was connected to the sensing FBG.

**Figure 7:**
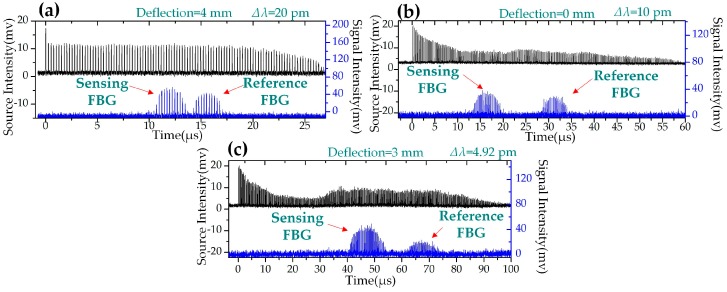
Waveforms of FBG strain sensing based on the WSSL with different sweep steps (*∆λ*). (**a**) ∆λ = 20 pm and the deflection is 4 mm. (**b**) ∆λ = 10 pm and the deflection is 0 mm. (**c**) ∆λ = 4.92 pm and the deflection is 3 mm.

**Figure 8:**
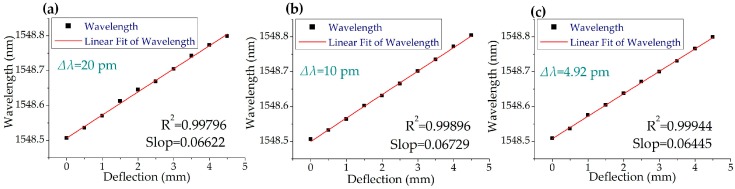
Interrogating results of FBG strain sensing with a reference FBG at different swept steps. (**a**) ∆λ = 20 pm. (**b**) ∆λ = 10 pm. (**c**) ∆λ = 4.92 pm.

Three strain-sensing waveforms are shown in Figure 7 as examples. In each graph, the black waveforms are the original pulses. The blue waveforms are the pulses reflected by the reference FBG and the sensing FBG. Figure 8 shows the interrogating results of FBG strain sensing at different swept steps. The central wavelength changed linearly with the increase in strain as expected. All the R-square values were higher than 0.997. The slopes of the linear fitting are about 0.0656 ± 0.002 nm/mm, which equaled to a strain sensitivity of 0.994 ± 0.03 pm/μɛ. As shown in Figure 8, the wavelength versus deflection linearity increased with the decrease in swept step (∆λ). 

### 3.2. Wavelength Interrogation of Dynamic Strain Sensing

In this experiment, the proposed wavelength step-swept laser source was applied to an FBG sensing system to measure dynamic strain. The reference FBG (the central wavelength was 1548.98 nm) was fixed on an anti-vibration table. The sensing FBG (center wavelength was 1549.48 nm) was stuck on the stage of a piezoelectric transducer (PZT) stack to allow the application of a dynamic periodic strain. A sinusoidal electrical signal generated by an arbitrary frequency generator (AFG) was applied to the PZT. The displacement of the PZT caused the strain variation that was applied to the sensing FBG. The dynamic strain sensing was interrogated by analyzing and calculating the changes in the central wavelength of the sensing FBG reflection signals in the time domain.

**Figure 9:**
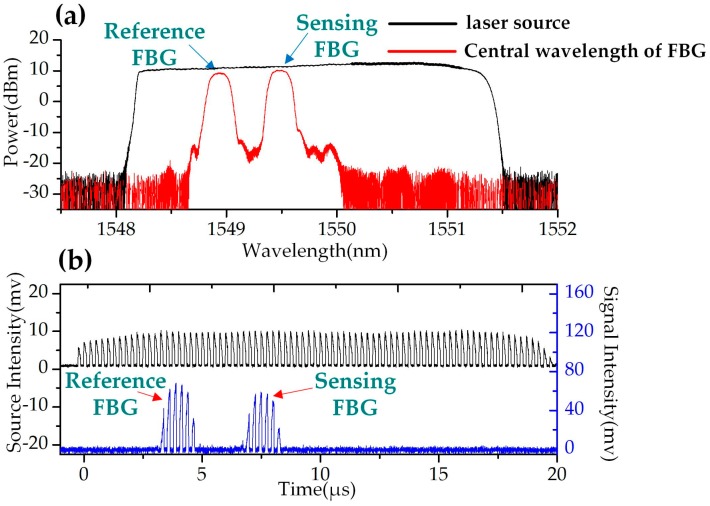
The reflection spectrum of reference and sensing FBGs in (**a**) the wavelength domain and (**b**) the time domain.

**Figure 10:**
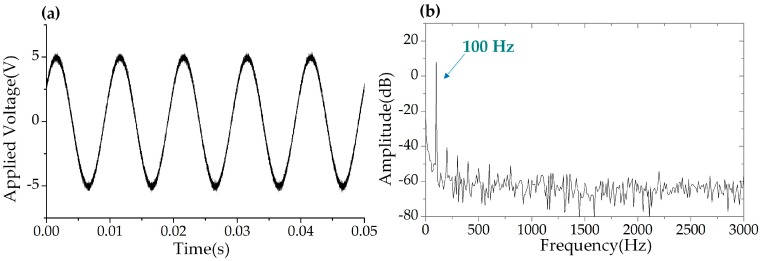
The driven signal applied to the piezoelectric transducer (PZT) stage. (**a**) The waveform in time domain. (**b**) The power spectral density of the FFT spectrum of (**a**).

**Figure 11:**
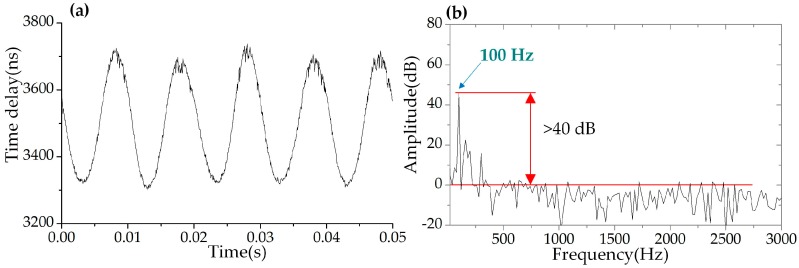
The interrogation result based on the WSSL at 40 kHz swept rate when the driven signal was 100 Hz. (**a**) The calculated time delay of the reflected pulses between the reference and sensing FBGs. (**b**) The power spectral density of the FFT spectrum of (**a**).

In this experiment, the central wavelength of the seed DFB laser was 1548.31 nm. The swept step was set to 40 pm (corresponding to 5 GHz) and the swept rate of the WSSL was set to 40 kHz. The bandwidth of the tunable filter was 3.5 nm and the swept range was 3.5 nm. The reflection spectrum in the wavelength domain and in the time domain of the two FBGs are shown in Figure 9. A sinusoidal electrical signal with frequency of 100 Hz and a voltage of 5 V was applied to the PZT stage. Figure 10 shows the driving waveform signal collected by the OSC and the power spectral density of the FFT spectrum. The periodic reflected signals of the FBGs were collected by a photodetector and recorded by a data acquisition (DAQ) card board with 200 Msamples/s and 8-bit resolution. The wavelength difference between the reference FBG and the sensing FBG was converted to a time delay of the reflected pulses. The interrogation results are shown in Figure 11. The time delay corresponding to the central wavelength difference between the two FBGs was a sinusoidal variation, shown in Figure 11a. The power spectral density of the FFT spectrum of Figure 11a is shown in Figure 11b. The low frequency noise between 100 Hz and 300 Hz was the white noise of the PZT actuator, as shown in Figure 10b. The SNR was estimated higher than 40 dB, and the frequency bandwidth was 20 Hz. The peak-to-peak amplitude of the dynamic strain was 61.26 με, calculated from Figure 11a. The RMS value of the applied strain was 21.66 με _rms_. The minimum detectable strain is determined by the background noise-level, which can be calculated by scaling the power spectral density of the noise by the square root of the bandwidth [4,16,17]. Therefore, the minimum detectable dynamic strain was calculated as 0.048 με/Hz^1/2^ when the SNR was 40 dB and the frequency was at 100 Hz.

**Figure 12:**
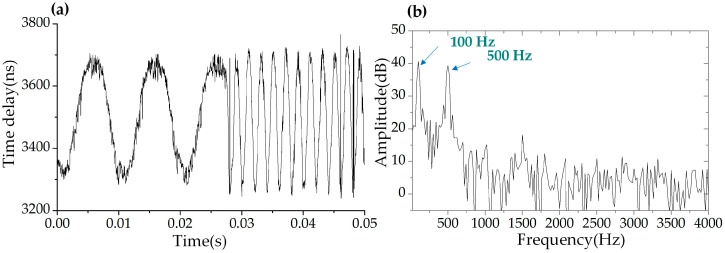
Dynamic response of the system. (**a**) The calculated time delay of the reflected pulses from the reference FBG and the sensing FBG when the frequency of the driven sinusoidal signal is abruptly changed from 100 Hz to 500 Hz. (**b**) The power spectral density of the FFT spectrum of (**a**).

In order to interrogate the dynamic strain in real-time, the driven sinusoidal signal applied to the PZT was abruptly changed from 100 Hz to 500 Hz during a transient time of about 25 μs. We used our WSSL system to record the dynamic response of the sensing FBG and the result were shown in Figure 12a. The time delay curve in the figure clearly displayed the period variation, which proved that this system could capture sudden changes in signal frequency. The interrogation result obtained by calculating the power spectral density of the FFT spectrum was shown in Figure 12b. There were two peaks at 100 Hz and 500 Hz, respectively, matching the frequency changes introduced in the driven signal. These results demonstrated that the dynamic strain can be interrogated by this system in real-time. 

## 4. Conclusions

We have proposed a wavelength step-swept source based on a recirculating frequency shifter loop, with which a fast FBG sensing system providing high interrogation linearity was realized. The wavelength of the output of the WSSL swept linearly (e.g., R-square value = 1 @ *f_RF_* = 10 GHz) in the time domain. Meanwhile, the swept rate could be tuned up to 99 kHz. The static strain sensing and the temperature sensing were successfully interrogated by the WSSL with different swept steps. The interrogation linearity of temperature and strain sensing were 0.99946 and 0.99944, respectively, with a 4.92 pm swept step. The sensitive of the interrogation results for the static strain and the temperature were 0.994 ± 0.03 pm/μɛ and 10.004 ± 0.1 pm/°C respectively. A dynamic periodic strain was also interrogated with the swept rate of the WSSL at 40 kHz. The SNR was over 40 dB and the bandwidth of the power spectral density of FFT spectrum was 20 Hz when the dynamic strain frequency was at 100 Hz. The calculated sensitivity was 0.048 με/Hz^1/2^. The dynamic response of the FBG successfully captured the sudden jump of the strain frequency from 100 Hz to 500 Hz with the WSSL system. It is expected that the dynamic strain in real-time can be interrogated by this system. For further experiments, a multi-wavelength laser will be adopted as the seed source to broaden the swept range for multiplexed FBGs interrogation without reducing the swept rate and linearity.
